# Socio-demographic and behavioural determinants of body mass index among an adult population in rural Northern Ghana: the AWI-Gen study

**DOI:** 10.1080/16549716.2018.1467588

**Published:** 2018-07-11

**Authors:** Engelbert Adamwaba Nonterah, Cornelius Debpuur, Godfred Agongo, Lucas Amenga-Etego, Nigel J. Crowther, Michèle Ramsay

**Affiliations:** a Navrongo Health Research Centre (NHRC), Navrongo, Ghana; b Julius Global Health, Julius Center for Health Sciences and Primary Care, University Medical Centre Utrecht, Utrecht University, Utrecht, The Netherlands; c Sydney Brenner Institute for Molecular Bioscience and Division of Human Genetics, School of Pathology, University of the Witwatersrand, Johannesburg, South Africa; d Department of Chemical Pathology, National Health Laboratory Service, University of the Witwatersrand, Johannesburg, South Africa

**Keywords:** Cardiometabolic diseases, socio-demographic and behavioural risk, pesticides, BMI, rural Northern Ghana

## Abstract

**Background**: Obesity and associated cardiometabolic diseases are increasing in urban sub-Saharan Africa due to a complex epidemiological and nutritional transition. Related data on rural communities is scarce.

**Objectives**: The study characterized the socio-demographic and behavioural factors influencing body mass index (BMI) among adults in rural Northern Ghana

**Methods**: A population-based cross-sectional study involving adults aged 40–60 years residing in the Kassena-Nankana districts was undertaken. Demographic, socio-economic and behavioural data were collected along with measures of anthropometry. We determined factors associated with BMI among women and men.

**Results**: A total of 2014 adults were studied. The median age was 51 (IQR 45–57) years and 54% were women. The prevalence of overweight/obesity was higher among women than men (18.4% vs. 7.2%; *p *< 0.001), whilst underweight was more prevalent in men (18.3% vs. 13.1%; *p *= 0.001). Participants with the highest level of education and a high household socio-economic status had higher BMIs than those in the lowest strata in both men (β = 0.074, *p = *0.028 and β = 0.072, *p *< 0.001, respectively) and women (β = 0.174, *p *= 0.001 and β = 0.109, *p *< 0.001, respectively). Men (β = −0.050; *p* < 0.001) and women (β = −0.073; *p* < 0.001) of the Nankana ethnic group had a lower BMI than the Kassena ethnic group. Among men, alcohol consumption (β = −0.021; *p* = 0.001) and smoking (β = −0.216; *p* < 0.001) were associated with lower BMI. Smokeless tobacco was associated with lower BMI among women. Pesticide exposure was associated with higher BMI (β = 0.022; *p* = 0.022) among men.

**Conclusion**: Age, sex, ethno-linguistic group and prevailing socio-demographic and behavioural factors within this rural community in Northern Ghana influence BMI. The observed positive association between pesticide use and BMI warrants further investigation.

## Background

Cardiometabolic diseases have become a major global health concern and are specifically increasing in sub-Saharan Africa (SSA) [1,2]. Countries in SSA are at different stages of the transition, with the majority moving from under- to over-nutrition [3–5]. This is largely due to the consumption of energy-rich foods and falling levels of energy expenditure [6].

The net effect of these transitions is an increasing incidence of deaths due to cardiometabolic diseases in both rural and urban Ghana [4,7]. The ensuing double burden of disease requires a deeper understanding of the prevailing factors in various communities that drive these emerging diseases.

The relative contribution of abnormal nutrition as an important risk factor for cardiometabolic disease-related morbidity and mortality is well characterized [8]. Whereas over-nutrition (manifested as overweight and obesity) is gaining much attention in the current complex epidemiological transition within most African countries, a substantial amount of under-nutrition (manifested as underweight) still persists in poorer communities across the continent [1]. Excess weight gain has been linked to type 2 diabetes, ischaemic heart disease, stroke, hypertensive heart disease, osteoarthritis, and cancers of the breast, colon, endometrium and kidney [9]. Evidence from verbal autopsy reports from the Navrongo Health and Demographic Surveillance System (NHDSS) point to the fact that cardiovascular and ischaemic heart diseases are among the top 10 causes of mortality in the Kassena-Nankana districts [10]. The overall contribution of malnutrition (both over-nutrition and under-nutrition) and other socio-demographic and lifestyle factors is therefore becoming more important in understanding the risk factors for the emerging threat of non-communicable diseases.

Body mass index (BMI), categorized as underweight (BMI < 18.5kg/m^2^), normal weight (BMI 18.5 to 24.9 kg/m^2^), overweight (BMI 25 to < 29.9 kg/m^2^) and obesity (BMI ≥ 30kg/m^2^) [2,11], remains an important proxy for assessing nutritional status in adults. It is a convenient measure of total body fat mass in resource constrained settings and has been demonstrated to correlate directly with co-morbidities and mortalities [6].

The Ghana national Demographic and Health Surveys (GDHS) and other studies have shown that the prevalence of obesity and overweight among adult non-pregnant women increased 2.5-fold in 10 years from 10% in 1993 to 25.3% in 2003 [12,13]. Recent estimates from poor urban settings in Accra, Ghana reported a higher prevalence of obesity (30.4%) among females [14]. It is, however, not clear whether the rising prevalence of obesity is homogenously distributed across the rural–urban divide. Few studies have attempted to investigate the effect of under-nutrition on cardiometabolic disease risk. The Non-communicable Disease Risk Factor Collaboration gave a global estimate of the trends in underweight recently. This study observed that underweight was still high in West, East and Central Africa and was associated with a slow decline over the past decade [1].

Major socio-demographic factors associated with adult nutritional status, measured using BMI as a proxy, include age, gender and socio-economic status [15–17]. Reported lifestyle factors include alcohol consumption, smoking [18,19] and physical inactivity [20]. The role of genetic factors in determining BMI has also been extensively reported in the literature [21,22]. Several cross-sectional and longitudinal studies in non-African countries have also observed a positive association between BMI and pesticide exposure [23–27]. However, this observation has not been previously reported in literature from SSA.

Only two previous studies have analysed the possible causes of obesity in a Ghanaian population. These investigations evaluated the effect of socio-demographic and behavioural factors on obesity and both were conducted in urban settings [14,28]. In view of the established influence of BMI on cardiovascular diseases (CVDs), which are a major contributor to morbidity and mortality, it is important to determine the prevalence of BMI categories and characterize the socio-demographic, behavioural and biological factors influencing their levels in this rural Ghanaian population.

In this study, nested in the H3Africa (Human Heredity and Health in Africa) AWI-Gen (Africa Wits-INDEPTH [International Network for the Demographic Evaluation of Populations and Their Health] partnership for Genomic studies) project [29], we report the prevalence of BMI categories ranging from underweight to obesity, and the influence of socio-demographic and behavioural factors on BMI among adults in the Kassena-Nankana districts in rural Northern Ghana. The age group selected for this study were 40–60 years old as the prevalence of obesity and associated cardiometabolic diseases rises during this period [15–17]. The current study will act as a baseline time point for a longitudinal analysis of such diseases in rural Ghana.

## Methods

### Study setting

**Figure 1.**
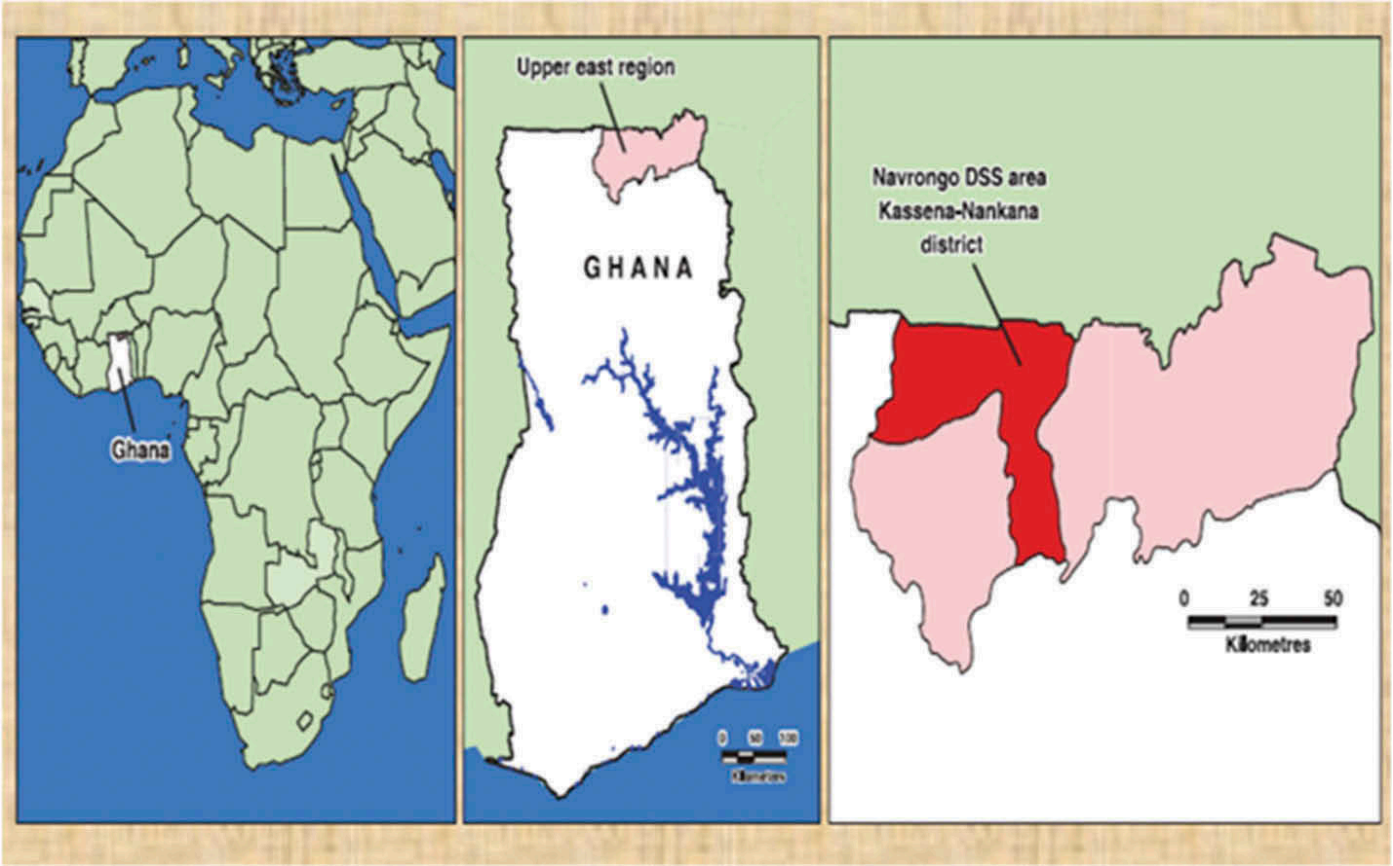
Maps showing Ghana in Africa, Kassena Nankana district in the upper east region and the Navrongo HDSS coverage area.

The study was conducted in the two Kassena-Nankana districts of Northern Ghana. The districts border Burkina Faso in the northeast and have a total land area of 1675km^2^ with a population density of 91.5 km^2^ (Figure 1) [30]. The districts form the coverage area of the Navrongo Health and Demographic Surveillance System (NHDSS) being carried out by the Navrongo Health Research Centre (NHRC), an INDEPTH Network pioneer member site. The NHDSS currently carries out surveillance on 165,000 individuals (52.3% females) in 32,000 households [31].

The study area is typical of many rural areas in sub-Saharan Africa where agriculture is predominantly the mainstay of the local economy with about 90% of the people being farmers [30]. The Tono irrigation dam (see Figure 1) located in the west zone of the district further serves as a good agricultural resource for dry season farming and a potential source of exposure of farmers to pesticides.

### Study design

This was a population-based cross-sectional study that recruited both men and women aged 40–60 years who had been resident within the study area for at least 10 years. Recruitment took place between February and October 2015. The communities within the study area were randomly sampled and the participants randomly selected using the NHDSS existing sampling frame, aiming for a sex and ethnic balance. This study was nested within the AWI-Gen study, which is a large pan-African investigation that aims to assess the genetic, physiological and environmental factors that contribute to BMI, body fat distribution and cardiometabolic disease risk factors in older (40–60 years old) African subjects in both rural and urban environments [29].

### Data collection

Trained research assistants conducted all interviews using a pretested comprehensive AWI-Gen questionnaire and carried out all measurements in this study. Interviews were conducted in English, Kassem and or Nankam languages based on the participants’ fluency.

Socio-demographic variables collected included age, sex, highest level of education attained, employment status, crowding in the household and household socio-economic status. Behavioural factors included smoking history, alcohol consumption, dietary history, physical activity and exposure to pesticides. The Global Physical Activity Questionnaire (GPAQ) [32] and the CAGE questionnaire [33] were incorporated into the main AWI-Gen questionnaire and used to assess physical activity and alcohol consumption respectively (see Supplementary material Table S3). Details of all data collection methods and measurements carried out in the study are described elsewhere [29] and are also presented as supplementary material (Table S3).

Measurement of weight and height. The participants, in light clothes and without shoes and jewellery, were made to stand vertically on a digital weighing scale and their weight was measured to the nearest 0.1 kilograms (Kg) using a calibrated standardized (Seca GmbH & Co. KG, Hamburg, Germany) weighing scale and according to standard practice. Their standing height was recorded to the nearest 0.1 millimetres (mm) using a stadiometer (Holtain, Crymych, Wales). The BMI was calculated from weight and height using the formula BMI = weight in kg/(height in m^2^) and subsequently categorized into underweight, normal weight, overweight and obesity according to World Health Organization (WHO) recommendations [2,11].

### Conceptual framework

**Figure 2.**
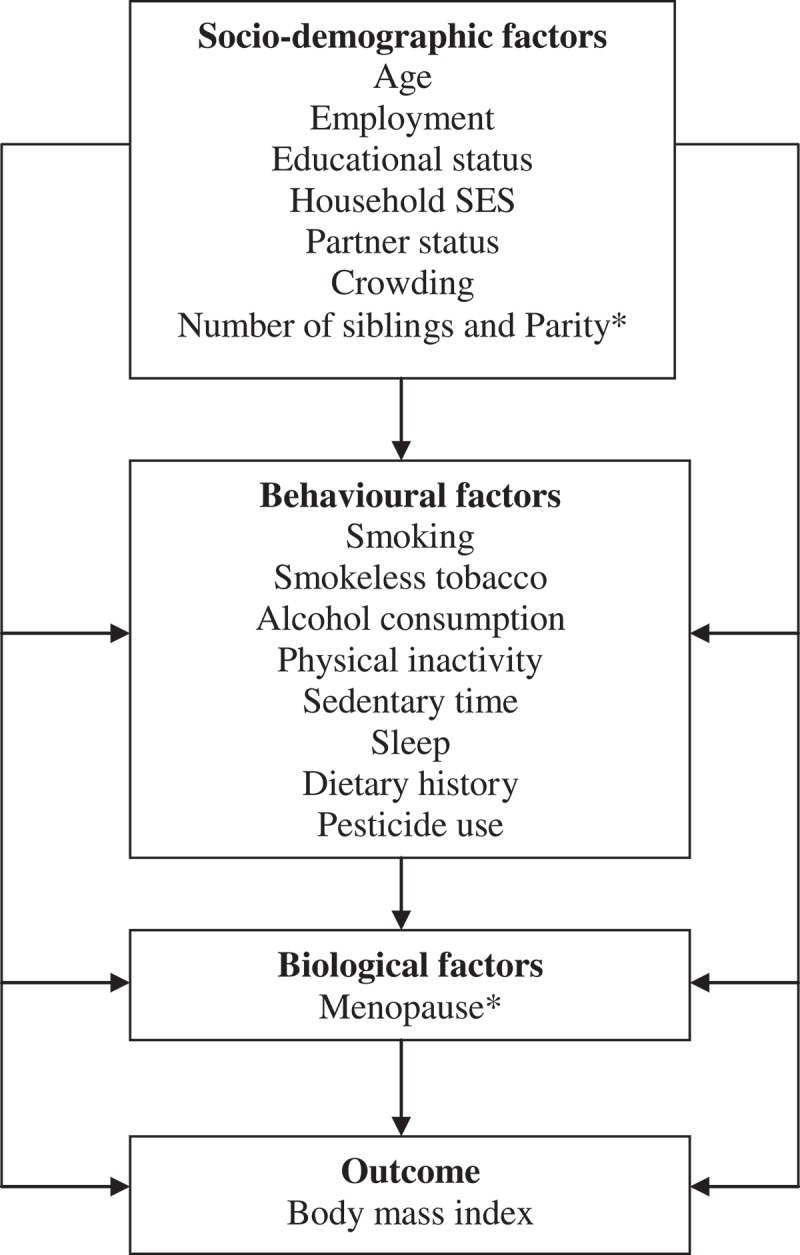
Conceptual framework for hierarchical regression analysis for men and women. *Factors peculiar to women only.

We sought to determine socio-demographic and behavioural factors that influence BMI among men and women. A hierarchical regression model was used to determine how each of these factors are independently associated with BMI and how they also interact with each other to influence BMI in both men and women. This method of data analysis is often used in studies where the determinants of disease are being sought, and where many possible risk factors are present and may influence the disease directly or via mediation of other factors [34]. This complex interaction of risk factors and their logical sequence in the models is normally depicted using a conceptual framework, as shown in Figure 2.

### Statistical analyses

All paper-based data were entered into a RedCap database [35,36]. The data were anonymized and 10% of the entries were checked for consistency between the paper and the electronic captured form as part of the quality control processes

Data in the RedCap data base were exported into Excel where further consistency checks and data cleaning were carried out. The final dataset was then exported into STATA version 13.1 MP (College Station, Texas 77845 USA) where all analyses were performed. All analyses were stratified by sex since there were significant sex differences for many parameters.

Basic descriptive statistics were presented as medians and interquartile range (IQR) for skewed continuous variables and frequencies and proportions for categorical variables. Mann-Whitney test (Rank sum test), a non-parametric test and Pearson’s Chi-squared were used to examine differences in medians and proportions respectively between men and women. The BMI categories and selected cardiometabolic risk factors were reported in proportions and 95% confidence intervals with Pearson’s Chi-squared test used to examine statistically significant differences in prevalence across selected groups. Body mass index was analysed as a categorical variable (underweight, normal weight, overweight and obese) and the prevalence of each of these categories across subgroups of cardiometabolic risk variables (i.e. age, alcohol consumption and pesticide use) was assessed.

Factors that influenced BMI in in both men and women were isolated using BMI as a continuous variable in hierarchical linear regression analysis. The outcome variable, BMI, was log-transformed to an approximate normal distribution. Model building followed a hierarchical conceptual framework (Figure 2) to control for confounding factors. All variables significant at *p* < 0.2 in an initial bivariable regression analysis with logBMI were included in the hierarchical model.

In the hierarchical modelling, we examined the influence of socio-demographic factors on BMI in regression model 1. The influence of behavioural factors on logBMI was determined in model 2, with adjustment for all the socio-demographic factors from model 1. Model 3 examined the influence of menopause on logBMI among women, with the inclusion of the variables from model 2. Therefore, two models were built for men while three models were built for women.

In building the models we checked for multicollinearity between related variables using the variance inflation factor (VIF). Variables with a VIF > 10 were dropped from the model. Akaike’s and Schwarz’s Bayesian information criteria (AIC and BIC respectively) post-estimation test were used to compare the fitted models, and the Breusch-Pagan/Cook-Weisberg test for homogeneity of variance was used to ensure residuals were approximately homoscedastic (*p *> 0.05 are considered normally distributed). We determine the cumulative effect of the blocks of factors on BMI through the magnitude of change in the variance explained by the model (Adjusted R^2^) in each stage of model building. The fitted models were also checked for model specifications (*p *> 0.05 are correctly specified) and omitted-variable bias using the Ramsey regression specification-error test (*p* > 0.05 have no omitted variable bias).

Within the hierarchical regression models for BMI, some variables were subdivided into three or more categories: i.e. ethnicity, partnership status, education status, household socio-economic status (SES), smoking history and alcohol consumption. For these variables, a post-estimation test was undertaken and a single p-value given to show the level of significance of the BMI trend across the categories.

## Results

### Summary statistics

Table 1 presents the socio-demographic characteristics of the 2014 participants in this study. Men and women differed in terms of socio-economic characteristics as follows: women constituted 54.1% of the sample and their median age (52 years; IQR 47–56) was significantly higher than that of the men (50 years; IQR 46–55); (*p* < 0.001). The Kassena ethnic group (51%) constituted half of the study population followed by the Nankana ethnic group (44%) and 5% were other minority ethnic groupings.

**Table 1.** Basic socio-demographic characteristics of the study cohort stratified by sex.

	Women, n = 1092 (54.2%)	Men, n = 924 (45.8%)	Total, N = 2014	*p*-value for sex differences
**Age in years**	52 (47–56)	50 (46–55)	51 (45–57)	< 0.001
**Age groups in years**				
40–44	145 (13.3)	171 (18.5)	316 (15.7)	< 0.001
45–49	273 (25.0)	242 (26.2)	515 (25.6)	
50–54	237 (21.7)	220 (23.8)	457 (22.7)	
55–60	436 (40.0)	290 (31.4)	726 (36.0)	
**Ethnicity**				
Kassena	551 (50.5)	477 (51.7)	1028 (51.0)	< 0.001
Nankana	457 (41.9)	427 (46.3)	884 (43.9)	
Others	83 (7.6)	19 (2.0)	102 (5.1)	
**Partnership status**				
Never married	5 (0.5)	15 (1.6)	20 (1.0)	< 0.001*
Currently married	694 (63.6)	787 (85.4)	1481 (73.6)	
Divorced/separated	392 (35.9)	120 (13.0)	512 (25.4)	
**Highest level of education**				
No formal education	843 (77.6)	570 (61.9)	1413 (70.4)	< 0.001
Primary education	177 (16.3)	206 (22.4)	383 (19.1)	
Secondary education	57 (5.3)	118 (12.8)	175 (8.7)	
Tertiary education	9 (0.8)	27 (2.9)	36 (1.8)	
**Employment status**				
Unemployed	429 (39.4)	321 (34.9)	750 (37.3)	0.039
Employed	659 (60.6)	599 (65.1)	1258 (62.7)	
**People-to-bedroom density**	2 (0.7–8)	2 (0.5–8)	2 (0.5–10)	0.073
**Household SES categories**				
Poorest	228 (20.9)	145 (15.7)	373 (18.5)	< 0.001
Very poor	208 (19.1)	152 (16.5)	360 (17.9)	
Poor	219 (20.1)	168 (18.2)	387 (19.2)	
Less poor	250 (22.9)	223 (24.2)	473 (23.5)	
Least poor	186 (17.1)	235 (25.5)	421 (20.9)	

Values presented as frequencies (n) and percentage (%) or median and interquartile range (IQR). Distribution of missing data as follows: partner status 1; highest level of education 7; employment status 6; *Fishers exact test used to examine sex differences since number of women who were never married was 5.

A large proportion of the study participants were married (73.6%) and a majority had no formal education (70.4%) with men more likely to have formal education than women. The proportion of participants engaged in some form of employment (63%) was significantly greater than those who were unemployed (37%).

Table 2 shows the behavioural and anthropometric characteristics of 2014 study participants. Behavioural factors, with the exception of dietary patterns, differed by gender. Males consumed more alcohol (*p* < 0.001) and smoked more tobacco (*p* < 0.001) than females. More than half of the study population was reportedly exposed to pesticides, and more men than women (*p <* 0.001) had been exposed. The population had a high physical activity level with men having higher levels of moderate-to-vigorous physical activity (MVPA) than women (*p* < 0.001). There was, however, no sex difference in fruit intake (*p* = 0.135), vegetable intake (*p* = 0.056) and intake of sugar-sweetened beverages (*p* = 0.514). Regarding anthropometric indices, women had a slightly higher median BMI (21.40 Kg/m^2^; IQR 15.09, 37.37) than men (20.57Kg/m^2^; IQR 13.97–36.47) though not significantly different (*p *= 0.091).

**Table 2.** Behavioural and anthropometric characteristics of the study cohort stratified by sex.

	Women, n = 1092 (54.2%)	Men, n = 924 (45.8%)	Total, N = 2014	*p*-value for sex differences
**Alcohol status**				
Never	231 (21.2)	71 (7.7)	302 (15.0)	< 0.001
Previous	426 (39.1)	252 (27.4)	678 (33.7)	
Current non-problematic	167 (15.3)	461 (50.1)	628 (31.3)	
Current problematic	265 (24.4)	137 (14.9)	402 (20.0)	
**Smoking status**				
Never	1052 (96.6)	332 (36.0)	1384 (68.8)	< 0.001
Previous	21 (1.9)	388 (42.0)	409 (20.3)	
Current	16 (1.5)	203 (22.0)	219 (10.9)	
**Smokeless tobacco**				
Never	967 (89.4)	816 (89.6)	1783 (89.5)	0.932
Ever/current	114 (10.6)	95 (10.4)	209 (10.5)	
**Exposure to pesticides**				
No	561 (51.5)	370 (40.1)	931 (46.3)	< 0.001
Yes	529 (48.5)	552 (59.9)	1080 (53.7)	
**Physical activity**				
MVPA minutes per week	240 (0–1440)	300 (0–1440)	245 (0–1440)	< 0.001
Average sleeping hours per night	8 (4–12)	8 (4–11)	8 (3–12)	0.445
**Dietary activity – median (IQR)**				
Fruit servings per day	1 (0–10)	1 (0–10)	1 (0–10)	0.135
Vegetables servings per day	3 (0–7)	3 (1–7)	3 (0–8)	0.056
Sugar-sweetened beverages (SSB)	0 (0–7)	0 (0–4)	0 (0–5)	0.514
**Anthropometric variables**				
Height	1.58 (1.41–1.79)	1.68 (1.16–1.89)	1.62 (1.16–1.93)	< 0.001
Weight	53.70 (34.90–98.60)	57.80 (40.00–95.00)	55.60 (34.90–105.40)	< 0.001
BMI in kg/m^2^	21.40 (15.09–37.37)	20.57 (13.97–36.47)	20.96 (13.89–43.06)	0.091

Values presented as frequencies (n) and percentage (%) or median and interquartile range (IQR). Distribution of missing data as follows: alcohol status 4; smokeless tobacco 22 and Exposure to pesticides 2.

### Prevalence of BMI categories by selected cardiometabolic risk factors

**Figure 3.**
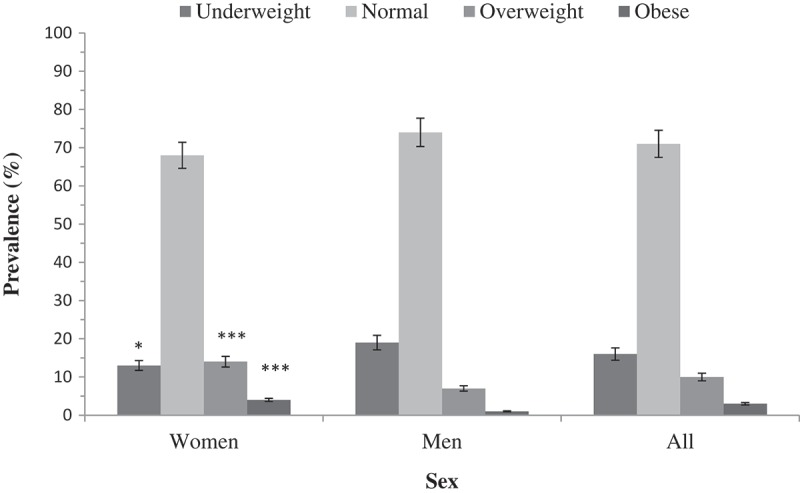
Prevalence of BMI categories stratified by sex. Data expressed as % with 95%CI and differences between women and men expressed as *p < 0.05, **p < 0.01 and ***p < 0.001.

The prevalence of the various categories of BMI by sex is presented in Figure 3 and the prevalence by selected cardiovascular risk factors such as age, alcohol consumption and exposure to pesticides presented in Table 3. These variables were chosen because of the known strong relationship of BMI with age and because alcohol consumption and pesticide use were very prevalent in this population. We observed that the prevalence of the various BMI categories differed by sex (*p* < 0.001), age (*p* < 0.001), alcohol consumption (*p* < 0.001) and exposure to pesticides (*p* < 0.001). A substantial proportion of the study population was underweight (15.5% [14.0, 17.2]) with more men (18.3% [15.9, 20.9]) being underweight compared to women (13.1% [11.3, 15.3]). Overweight and obesity were higher among women (14.2% [12.3, 16.4] and 4.2% [3.2, 5.6], respectively) than men (6.0% [4.6, 7.7] and 1.2% [0.7, 2.1], respectively) (see Figure 3).

**Table 3.** Prevalence of the various categories of BMI (in kg/m^2^) by selected cardiometabolic risk factors among women and men in Navrongo.

	Underweight	Normal weight	Overweight	Obesity	*P-value
**Women**					
**Age groups in years**					
40–44	6.2 [3.3,11.5]	71.0 [63.1,77.8]	17.2 [11.9,24.3]	5.5 [2.8,10.6]	0.001
45–49	7.7 [5.1,11.5]	70.7 [65.0,75.8]	15.8 [11.9,20.6]	5.9 [3.6,9.4]	
50–54	13.1 [9.3,18.0]	70.9 [64.8,76.3]	12.7 [9.0,17.5]	3.4 [1.7,6.6]	
55–60	18.8 [15.4,22.8]	64.9 [60.3,69.3]	13.1 [10.2,16.6]	3.2 [1.9,5.4]	
**Alcohol consumption**					
Never	11.7 [8.1,16.5]	61.0 [54.6,67.1]	20.8 [16.0,26.5]	6.5 [4.0,10.5]	0.049
Previous	13.1 [10.3,16.7]	71.1 [66.6,75.2]	12.7 [9.8,16.2]	3.1 [1.8,5.2]	
Current non-problematic	12.0 [7.9,17.8]	70.7 [63.3,77.1]	13.2 [8.8,19.2]	4.2 [2.0,8.5]	
Current problematic	15.1 [11.3,19.9]	69.1 [63.2,74.3]	11.7 [8.3,16.2]	4.2 [2.3,7.3]	
**Exposure to Pesticides**					
No	18.4 [15.4,21.8]	66.1 [62.1,69.9]	12.1 [9.7,15.1]	3.4 [2.2,5.3]	<0.001
Yes	7.6 [5.6,10.1]	71.1 [67.1,74.8]	16.4 [13.5,19.9]	4.9 [3.4,7.1]	
**Men**					
**Age groups in years**					
40–44	10.5 [6.7,16.1]	77.8 [70.9,83.4]	9.9 [6.3,15.4]	1.8 [0.6,5.3]	0.003
45–49	17.8 [13.4,23.1]	73.6 [67.6,78.7]	7.9 [5.1,12.0]	0.8 [0.2,3.2]	
50–54	19.1 [14.4,24.8]	73.6 [67.4,79.0]	5.0 [2.8,8.8]	2.3 [0.9,5.3]	
55–60	22.8 [18.3,27.9]	74.1 [68.8,78.9]	2.8 [1.4,5.4]	0.3 [0.0,2.4]	
**Alcohol consumption**					
Never	4.2 [1.4,12.3]	77.5 [66.3,85.7]	15.5 [8.8,25.9]	2.8 [0.7,10.6]	<0.001
Previous	13.5 [9.8,18.3]	80.2 [74.8,84.6]	5.6 [3.3,9.2]	0.8 [0.2,3.1]	
Current non-problematic	21.9 [18.4,25.9]	72.5 [68.2,76.3]	4.8 [3.2,7.1]	0.9 [0.3,2.3]	
Current problematic	21.9 [15.8,29.6]	70.1 [61.9,77.1]	5.8 [2.9,11.2]	2.2 [0.7,6.6]	
**Exposure to Pesticides**					
No	27.8 [23.5,32.6]	67.0 [62.1,71.6]	4.1 [2.5,6.6]	1.1 [0.4,2.8]	<0.001
Yes	12.0 [9.5,14.9]	79.5 [76.0,82.7]	7.2 [5.4,9.7]	1.3 [0.6,2.6]	

Values presented as proportions (%) and 95% confidence intervals (CI). *Fisher’s exact test used to derive p-values because some of the cells had less than 5 observations

The older participants were more likely to be underweight while the younger age groups were more likely to be overweight and obese. Participants who had never consumed alcohol had the highest prevalence of overweight and obesity whilst underweight was highest among participants who were current non-problematic or current problematic alcohol consumers. Women and men who were exposed to pesticides had a higher prevalence of overweight (4.9% and 7.4% respectively) compared to those not exposed (3.4% and 4.1% respectively) and a similar trend was observed for obesity (see Table 3).

### Factors associated with BMI

In the unadjusted bivariable analyses, higher age, tertiary education, higher socio-economic status (SES), use of sugar-sweetened beverages and exposure to pesticides were associated with higher BMI among both men and women. Men and women of the Nankana ethnic group and who were current problematic alcohol users were likely to have lower BMI. Former use of tobacco and longer sleep duration (see Supplementary material; Tables 1a & 1b) were likely to result in lower BMI in both genders.

Socio-demographic, behavioural and biological factors that influence logBMI among men and women are presented in Table 4. Models 1 and 2 are presented as supplementary material (Tables S2a, S2b, S3a and S3b). In model 1 we included only the socio-demographic variables. Age, ethnicity, educational status and household socio-economic status were all significantly associated with BMI. Thus, older participants had lower BMI among men and women. The Nankana ethnic group in both genders had lower BMI compared to Kassena while other ethnicities in the population had higher BMI. Higher level of education and higher household socio-economic status resulted in higher BMI among men and women respectively.

**Table 4.** Factors associated with logBMI among women and men determined through hierarchical multivariable linear regression analysis.

	Women		Men	
	Adjusted β-coefficients [95%CI]	*p*-value	Adjusted β-coefficients [95%CI]	*p*-value
Age	−0.004 [−0.005, −0.002]	< 0.001	−0.003 [−0.004, −0.001]	< 0.001
**Ethnicity**				
Kassena	ref	ref	ref	ref
Nankana	−0.073 [−0.093, −0.054]	< 0.001	−0.050 [−0.068, −0.033]	< 0.001
Others	0.011 [−0.024, 0.047]		0.075 [0.016, 0.134]	
**Partnership status**				
Single	-	-	−0.063 [−0.129, 0.003]	0.005
Currently married	-	-	ref	ref
Divorced/separated	-	-	−0.037 [−0.063, −0.011]	
**Educational status**	-	-		
No formal education	ref	ref	ref	ref
Primary education	0.022 [−0.004, 0.047]	0.001	0.005 [−0.016, 0.026]	0.028
Secondary education	0.067 [0.022, 0.112]		0.008 [0.005, 0.039]	
Tertiary education	0.174 [0.052, 0.296]		0.074 [0.022, 0.126]	
**Household SES**				
Poorest	ref	ref	ref	ref
Very poor	−0.008 [−0.037, 0.021]	< 0.001	−0.011 [−0.042, 0.017]	< 0.001
Poor	0.017 [−0.011, 0.047]		0.025 [−0.004, 0.054]	
Less poor	0.037 [0.008, 0.065]		0.008 [−0.019, 0.036]	
Least poor	0.109 [0.077, 0.141]		0.072 [0.043, 0.101]	
**Smokeless tobacco**				
No	ref	ref	-	-
Yes	−0.039 [−0.069, −0.009]	0.009	-	-
**Smoking history**			-	-
Never	-	-	ref	ref
Previous	-	-	−0.092 [−0.158, −0.026]	< 0.001
Current	-	-	−0.216 [−0.097, 0.054]	
**Alcohol consumption**				
Never	-	-	0.008 [−0.031, 0.048]	0.001
Previous	-	-	ref	ref
Current non-problematic	-	-	−0.005 [−0.035, −0.001]	
Current problematic	-	-	−0.021 [−0.042, −0.003]	
**Sedentary time**	0.004 [0.001, 0.029]	0.040	0.045 [0.019, 0.074]	0.026
**Average sleep duration**	−0.008 [0.015, −0.001]	0.022	−0.002 [−0.004, −0.001]	0.018
**Pesticide use**				
No	-	-	ref	ref
Yes	-	-	0.022 [0.003, 0.042]	0.022

In model 2, we report varied associations between behavioural factors and BMI among men and women. In women (note that model 2 in Table S2a and the final model 3, shown in Table 4, differ only in terms of the inclusion of menopause status in the latter model), higher sedentary time (β-coefficient = 0.004 [0.001, 0.029]) or longer sleep duration (−0.008 [−0.015, −0.001]) resulted in higher or lower BMI, respectively. In model 2 (final model for men shown in Table S2b and Table 4) similar effects on BMI were observed in men as for women for sleep duration (−0.002 [−0.004, −0.001] and sedentary time (0.045 [0.019, 0.074]). In addition, both previous and current smoking among men caused a decrease in BMI by 9.2% (−0.092 [−0.158, −0.026]) and 21.6% (−0.216 [−0.097, 0.054]) respectively, relative to those who never smoked. The use of smokeless tobacco products such as snuff and chewing tobacco was associated with a reduced BMI among women (−0.039 [−0.069, −0.009]) but there was no association with BMI among men (*p* = 0.611). Men who had never consumed alcohol were more likely to have a higher BMI (0.008 [−0.031, 0.048]) compared to those who were currently drinking. Current non-problematic consumption of alcohol resulted in 0.5% (−0.005 [−0.035, −0.001]) reduction in BMI while current problematic consumption of alcohol resulted in 2.1% (−0.021[−0.042, −0.005]) reduction in BMI among men when compared to those who never consumed alcohol. Men who reported to have been exposed to pesticides had 1.3% higher BMI (0.013 [0.005, 0.033]) compared to unexposed men.

In model 2 (final model for men), the AIC/BIC were smaller (−1036.72/−898.51) than in model 1 (−1136.74/−1060.05) and this model was correctly specified (p = 0.104) with no omitted variable bias (*p* = 0.678). The adjusted R^2^ was also improved from 0.163 to 0.201. Model 3 (final model for women) had a smaller AIC/BIC (−587.81/−449.46) compared to models 1 and 2 (−967.74/−884.46 and −947.20/−805.82 respectively) and was correctly specified (*p* = 0.077) with no variable bias (*p* = 0.149). The adjusted R^2^ also improved from 0.178 in model 1 to 0.189 in model 2 and 0.193 in model 3. The mean VIF in the final hierarchical models for women and men were 1.25 and 1.24 respectively.

## Discussion

Sub-Saharan Africa is currently undergoing a complex epidemiological, demographic and nutritional transition that produces a huge double burden of diseases. Understanding factors that influence the BMI of adults as a measure of nutritional status is one key step in addressing the problem. To our knowledge no study has explored the contribution of socio-demographic and behavioural factors to BMI levels among adults in rural Northern Ghana. While obesity is on the increase due to changing dietary patterns and lifestyle, underweight, signifying under-nutrition, is still highly prevalent in rural communities. Our study reports various socio-demographic and behavioural factors that influence BMI in both women and men in a rural population in Northern Ghana.

We observed a high prevalence of various risk factors for cardiometabolic diseases in this population. Older participants and those who currently consume alcohol (both non-problematic and problematic drinking patterns) were likely to be underweight. This may reflect the micronutrient deficiencies that are associated with alcohol consumption [19]. Fat mass is known to decrease in much older subjects [37] and may contribute to the lower BMI associated with higher age observed in this study. The population consumed little fruit and vegetables per day but had a high consumption of sugar-sweetened beverages compared to international recommendations [37].

Even though overweight and obesity were relatively low in our population compared to other African countries further along the epidemiological transition, women were more likely to be overweight or obese. The sex-specific pattern of overweight and obesity reflects the general distribution reported across Africa [8,38] and in previous reports from Ghana [6,39,40]. The roles of endogenous and exogenous steroid exposure and biological differences in body fat distribution have been used to explain gender differences in overweight and obesity [16].

We observed that men and women who belong to the Nankana ethnic group were likely to have a lower BMI than the other ethno-linguistic groups. This was not due to differences in socio-demographic or behavioural variables as this relationship was maintained in the final hierarchical regression models. These results suggest that ethnic differences in BMI may be related to other unmeasured variables which may include genetic factors.

Men and women who had attained a higher education or had higher household income were likely to have a higher BMI. The latter observation mirrors reports from other low- and middle-income countries (LMIC) [6,41,42] but differs from many findings emanating from western countries which have often reported higher BMI among families with lower SES [43]. The data from LMICs may be due to a high purchasing power of those with higher SES who undergo a faster nutritional transition onto energy-dense foods. Such individuals are likely to also have the ability to afford automobiles and are more likely to be physically inactive. Recent studies among Ghanaians had also found a strong association between higher household income levels, higher educational status and type 2 diabetes (T2D) and obesity [44]. Possible reasons include the fact that higher education is associated with a higher likelihood of engaging in white-collar or more sedentary jobs [6]. The subsequent inactivity associated with these jobs will likely result in increased BMI. Some studies in Ghana have alluded to the fact that socioculturally, a large body size among females is perceived as a sign of affluence [27], and women with higher education are likely to be affluent and have more exposure to energy-dense foods. The observed association of sedentary lifestyle with higher BMI among men and women is largely consistent with other studies both outside [1] and within Africa [39,45] and Ghana [41,42].

Regarding smoking history, men who smoked previously or were currently smoking reported lower BMI compared to those who had no history of smoking. These findings are consistent with findings from previous studies [46,47]. This negative association may be attributed to reduced caloric intake due to a central effect of smoking, impaired smell or taste, a change in food preference, or a direct metabolic effect on the absorption or storage of calories, or an increase in energy expenditure [48]. The association between smokeless tobacco use and increased risk of cardiometabolic-related morbidities and mortalities has been well characterized [49–51]. However, in our study, smokeless tobacco use was associated with a lower BMI in women. Similar findings have previously been reported among native Yup’ik Alaskan male and female adult population [52], among Swiss men [53] and in African populations [54].

We observed that longer sleep duration was associated with lower BMI in both men and women. This is consistent with similar studies which report that older participants with longer sleep duration were likely to have lower BMI [55]. Sedentary time, however, was associated with higher BMI among both men and women. This observation is also consistent with other studies [56]. Furthermore, increased physical activity has been extensively reported to result in reduction in BMI [57–59]. However, despite the fact that this ageing population was an active one, we observed no association of physical activity with BMI.

Our data also showed the significant contribution of alcohol to adult under-nutrition and underweight especially among the male population. The association between alcohol consumption and weight gain remains controversial. The energy derived from the high calorie content of alcohol suggests that excessive alcohol intake will lead to weight gain and a French study has reported this observation [60]. However, alcohol intake may not systematically increase BMI as this relationship is dependent on variations and patterns of alcohol intake, energy expenditure due to physical activity and general nutritional status. Similar to our findings, other studies have observed that moderate and hazardous alcohol intake is associated with lower BMI [61]. It has also been reported that chronic alcoholics have a lower fat mass than non-alcoholics [62]. The potential influence of alcohol abuse on cardiovascular-related morbidity and mortality remains to be elucidated in this population.

Pesticide exposure was higher among men in our study population and was found in regression analyses to be associated with higher BMI among men. Cross-sectional [24,26,27,63] and longitudinal [23,25] studies have reported similar findings. This observation in our population is of great concern and a potential driver for obesity in this population due to the high exposure to pesticides. Pesticides contain chemical compounds that are widely considered as obesogenic and endocrine disruptors. These compounds are reported to modulate lipid metabolism, hormone and neurotransmitter levels and adipogenesis and thus cause weight gain [23–27,63].

### Limitations

The behavioural data was collected based on self-reported responses from the participants and these could not be independently verified and may lead to biased estimates of our observed associations. The cross-sectional nature of this study comes with inherent limitations as causality cannot be established.

### Strengths

Very few studies in SSA have evaluated socio-demographic and behavioural factors associated with BMI in a large population of rural adults. Therefore, the results of this study provide important new information on the environmental factors influencing BMI in a large rural African population. The findings provide baseline information for a longitudinal cohort study on BMI in rural Northern Ghanaian adults who can easily be followed up using the NHDSS platform. The study also gives information on the prevalent risk factors that contribute to the observed rising mortalities due to cardiometabolic diseases reported from verbal autopsy data in the study area. It also generates further research questions into the potential effects of some of the observed risk factors on cardiovascular co-morbidities.

## Conclusion

The burden of risk factors for cardiometabolic diseases among adults in this rural population is high, especially among men. However, unlike other African populations further along the epidemiological transition, this ageing population from rural Ghana does not have a high prevalence of overweight and obesity. Interestingly, pesticide use in this predominantly farming community is high and correlates positively with obesity. Farmers may therefore benefit from an educational intervention based on the use of personal protective gear when working with pesticides.

## Paper context

Research into obesity within rural Ghana is scarce except for an ongoing verbal autopsy programme within the NHDSS which demonstrates rising levels of CVD-related deaths (unpublished). This study is the first to report on the socio-demographic and behavioural factors influencing BMI among adults in rural Northern Ghana. The data suggests that improvements in socio-demographic status within this population may be accompanied by rising levels of obesity and will require developing appropriate monitoring and intervention programmes.

## References

[1] Forouzanfar MH, Afshin A, Alexander LT, et al. Global, regional, and national comparative risk assessment of 79 behavioural, environmental and occupational, and metabolic risks or clusters of risks, 1990-2015: a systematic analysis for the Global Burden of Disease Study 2015. Lancet. 2016;388:1659–12. 27733284 10.1016/S0140-6736(16)31679-8 PMC5388856

[2] World Health Organization. Obesity: preventing and managing the global epidemic. Report of a WHO consultation. World Health Organ Tech Rep Ser. 2000;894:i-xii,1–253. 11234459

[3] Kuate Defo B. Demographic, epidemiological, and health transitions: are they relevant to population health patterns in Africa? Glob Health Action. 2014;7:22443. 24848648 10.3402/gha.v7.22443 PMC4028929

[4] Bawah A, Houle B, Alam N, et al The evolving demographic and health transition in four low- and middle-income countries: evidence from four sites in the INDEPTH network of longitudinal health and demographic surveillance systems. PLoS One. 2016;11:e0157281. 27304429 10.1371/journal.pone.0157281 PMC4909223

[5] Santosa A, Byass P Diverse empirical evidence on epidemiological transition in low- and middle-income countries: population-based findings from INDEPTH network data. PLoS One. 2016;11:e0155753. 27187781 10.1371/journal.pone.0155753 PMC4871496

[6] Ofori-Asenso R, Agyeman AA, Laar A, et al Overweight and obesity epidemic in Ghana—a systematic review and meta-analysis. BMC Public Health. 2016;16:1239. 27938360 10.1186/s12889-016-3901-4 PMC5148846

[7] Agyei-Mensah S, de-Graft Aikins A Epidemiological transition and the double burden of disease in Accra, Ghana. J Urban Health. 2010;87:879–897. 20803094 10.1007/s11524-010-9492-y PMC2937133

[8] NCD Risk Factor Collaboration (NCD-RisC) - African Working Group. Trends in obesity and diabetes across Africa from 1980 to 2014: an analysis of pooled population-based studies. Int J Epidemiol. 2017;46:1421–1432. 28582528 10.1093/ije/dyx078 PMC5837192

[9] Rodgers A, Ezzati M, Vander Hoorn S, et al Distribution of major health risks: findings from the global burden of disease study. PLoS Med. 2004;1:e27. 15526049 10.1371/journal.pmed.0010027 PMC523844

[10] Ansah P, Debpuur C, Akazili J, et al Validation of causes-of-death using verbal autopsy data collected from navrongo health and demographic surveillance system in Ghana 2007–2011. MEASURE Evaluation Project. Navrongo, Ghana. 2014 Available at http://www.navrongo-hrc.org/.

[11] Ferro-Luzzi A, Garza C, Haas J, et al Physical Status: the use and interpretation of anthropometry. Geneva: World Health Organisation; 1995.

[12] Biritwum RB, Gyapong J, Mensah G The epidemiology of obesity in Ghana. Ghana Med J. 2005;39:82–85. 17299549 PMC1790818

[13] Ghana Statistical Service (GSS), Ghana Health Service (GHS), and ICF International. Ghana Demographic and Health Survey 2014 Rockville, Maryland, USA: GSS, GHS, and ICF International; 2015.

[14] Dake FA, Thompson AL, Ng SW, et al The local food environment and body mass index among the urban poor in Accra, Ghana. J Urban Health. 2016;93:438–455. 27091736 10.1007/s11524-016-0044-y PMC4899328

[15] Amugsi DA, Dimbuene ZT, Bakibinga P, et al Dietary diversity, socioeconomic status and maternal body mass index (BMI): quantile regression analysis of nationally representative data from Ghana, Namibia and Sao Tome and Principe. BMJ Open. 2016;6:e012615. 10.1136/bmjopen-2016-012615 PMC5051549 27678544

[16] Lovejoy JC, Sainsbury A Sex differences in obesity and the regulation of energy homeostasis. Obes Rev. 2009;10:154–167. 19021872 10.1111/j.1467-789X.2008.00529.x

[17] Kodaman N, Aldrich MC, Sobota R, et al Cardiovascular disease risk factors in ghana during the rural-to-urban transition: a cross-sectional study. PLoS One. 2016;11:e0162753. 27732601 10.1371/journal.pone.0162753 PMC5061429

[18] Kingue S, Rakotoarimanana S, Rabearivony N, et al Prevalence of selected cardiometabolic risk factors among adults in urban and semi-urban hospitals in four sub-Saharan African countries. Cardiovasc J Afr. 2017;28:147–153. 27701489 10.5830/CVJA-2016-072 PMC5558135

[19] French MT, Norton EC, Fang HAI, et al Alcohol consumption and body weight. Health Econ. 2010;19:814–832. 19548203 10.1002/hec.1521 PMC3082959

[20] Fares D, Barbosa AR, Borgatto AF, et al Fatores associados ao estado nutricional de idosos de duas regiões do Brasil. Revista da Associação Médica Brasileira. 2012;58:434–441. 22930021

[21] Ajayi IO, Adebamowo C, Adami HO, et al Urban-rural and geographic differences in overweight and obesity in four sub-Saharan African adult populations: a multi-country cross-sectional study. BMC Public Health. 2016;16:1126. 27793143 10.1186/s12889-016-3789-z PMC5084330

[22] Skrypnik K, Suliburska J, Skrypnik D, et al The genetic basis of obesity complications. Acta Sci Pol Technol Aliment. 2017;16:83–91. 28362475 10.17306/J.AFS.2017.0442

[23] Andreotti G, Hou L, Beane Freeman LE, et al Body mass index, agricultural pesticide use, and cancer incidence in the agricultural health study Cohort. Cancer Causes Control. 2010;21:1759–1775. 20730623 10.1007/s10552-010-9603-9 PMC2962760

[24] Elobeid MA, Padilla MA, Brock DW, et al Endocrine disruptors and obesity: an examination of selected persistent organic pollutants in the NHANES 1999-2002 data. Int J Environ Res Public Health. 2010;7:2988–3005. 20717554 10.3390/ijerph7072988 PMC2922741

[25] LaVerda NL, Goldsmith DF, Alavanja MCR, et al Pesticide exposures and body mass index (BMI) of pesticide applicators from the agricultural health study. J Toxicol Environ Health. 2015;78:1255–1276. 10.1080/15287394.2015.1074844 26479458

[26] Lee DH, Steffes MW, Sjodin A, et al Low dose organochlorine pesticides and polychlorinated biphenyls predict obesity, dyslipidemia, and insulin resistance among people free of diabetes. PLoS One. 2011;6:e15977. 21298090 10.1371/journal.pone.0015977 PMC3027626

[27] Kanazawa A, Miyasita C, Okada E, et al Blood persistent organochlorine pesticides in pregnant women in relation to physical and environmental variables in The Hokkaido study on environment and children’s health. Sci Total Environ. 2012;426:73–82. 22503674 10.1016/j.scitotenv.2012.02.073

[28] Benkeser RM, Biritwum R, Hill AG Prevalence of overweight and obesity and perception of healthy and desirable body size in urban, Ghanaian women. Ghana Med J. 2012;46:66–75. 22942454 PMC3426384

[29] Ramsay M, Crowther NJ, Tambo E, et al H3Africa AWI-Gen Collaborative Centre: a resource to study the interplay between genomic and environmental risk factors for cardiometabolic diseases in four sub-Saharan African countries. Global Health, Epidemiology Genomics. 2016;1:1–13. 10.1017/gheg.2016.17 PMC5732578 29276616

[30] Binka FN, Ngom P, Phillips JF, et al Assessing population dynamics in a rural African society: the navrongo demographic surveillance system. J Biosoc Sci. 1999;31:375–391.

[31] Oduro AR, Wak G, Azongo D, et al Profile of the navrongo health and demographic surveillance system. Int J Epidemiol. 2012;41:968–976. 22933645 10.1093/ije/dys111

[32] Bull FC, Maslin TS, Armstrong T Global physical activity questionnaire (GPAQ): nine country reliability and validity study. J Phys Act Health. 2009;6:790–804. 20101923 10.1123/jpah.6.6.790

[33] Mayfield D, MCleod G, Hall P The CAGE questionnaire: validation of a new alcoholism screening instrument. Am J Psychiatry. 1974;131:1121–1123. 4416585 10.1176/ajp.131.10.1121

[34] Victora CG, Huttly SR, Fuchs SC, et al The role of conceptual frameworks in epidemiological analysis: a hierarchical approach. Int J Epidemiol. 1997;26:224–227. 9126524 10.1093/ije/26.1.224

[35] Harris PA, Taylor R, Thielke R, et al Research Electronic Data Capture (REDCap) - A metadata-driven methodology and workflow process for providing translational research informatics support. J Biomed Inform. 2009;42:377–381. 18929686 10.1016/j.jbi.2008.08.010 PMC2700030

[36] Klipin M, Mare I, Hazelhurst S, et al The process of installing REDCap, a web based database supporting biomedical research: the first year. Appl Clin Inform. 2014;5:916–929. 25589907 10.4338/ACI-2014-06-CR-0054 PMC4287671

[37] Hickson M Malnutrition and ageing. Postgrad Med J. 2006;82:2–8. 16397072 10.1136/pgmj.2005.037564 PMC2563720

[38] Micklesfield LK, Lambert EV, Hume DJ, et al Socio-cultural, environmental and behavioural determinants of obesity in black South African women. Cardiovasc J Afr. 2013;24:369–375. 24051701 10.5830/CVJA-2013-069 PMC3896104

[39] Abubakari AR, Lauder W, Agyemang C, et al Prevalence and time trends in obesity among adult West African populations: a meta-analysis. Obes Rev. 2008;9:297–311. 18179616 10.1111/j.1467-789X.2007.00462.x

[40] Ofori-Asenso R, Garcia D Cardiovascular diseases in Ghana within the context of globalization. Cardiovasc Diagn Ther. 2016;6:67–77. 26885494 10.3978/j.issn.2223-3652.2015.09.02 PMC4731582

[41] Amoah AG Obesity in adult residents of Accra, Ghana. Ethn Dis. 2003;13:S97–S101. 13677422

[42] Appiah CA, Steiner-Asiedu M, Otoo GE Predictors of overweight/obesity in urban Ghanaian women. Int J Clin Nutr. 2014;2:60–68.

[43] Pampel FC, Denney JT, Krueger PM, et al economic development: a test of the reversal hypothesis. Soc Sci Med. 2012;74:1073–1081. 22341204 10.1016/j.socscimed.2011.12.028 PMC3298576

[44] Addo J, Agyemang C, de-Graft Aikins A, et al Association between socioeconomic position and the prevalence of type 2 diabetes in Ghanaians in different geographic locations: the RODAM study. J Epidemiol Community Health. 2017;71:633–639. 28348205 10.1136/jech-2016-208322 PMC5485755

[45] Agyemang C, Meeks K, Beune E, et al Obesity and type 2 diabetes in sub-Saharan Africans – is the burden in today’s Africa similar to African migrants in Europe? The RODAM study. BMC Med. 2016;14:166. 27769239 10.1186/s12916-016-0709-0 PMC5075171

[46] Dare S, Mackay DF, Pell JP Relationship between smoking and obesity: a cross-sectional study of 499,504 middle-aged adults in the UK general population. PLoS ONE. 2015;10:e0123579. 25886648 10.1371/journal.pone.0123579 PMC4401671

[47] Tan AKG, Yen ST, Feisul MI The association between smoking and body mass index: results from a national sample of Malaysian adults. J Public Health. 2013;21:403–412.

[48] Berlin I Endocrine and metabolic effects of smoking cessation. Curr Med Res Opin. 2009;25:527–534. 10.1185/03007990802707626 28925757

[49] Lee PN Circulatory disease and smokeless tobacco in Western population: a review of the evidence. Int J Epidemiol. 2007;36:789–804. 17591642 10.1093/ije/dym039

[50] Critchley JA, Unal B Is smokeless tobacco a risk factor for coronary heart disease? A systematic review of epidemiological studies. Eur J Cardiovasc Prev Rehabil. 2004;11:101–112. 15187813 10.1097/01.hjr.0000114971.39211.d7

[51] Janzon E, Hedblad B Swedish snuff and incidence of cardiovascular disease. A population-based cohort study. BMC Cardiovasc Disord. 2009;9:21. 19473535 10.1186/1471-2261-9-21 PMC2695419

[52] Ryman TK, Boyer BB, Hopkins SE, et al Association between iq’mik smokeless tobacco use and cardiometabolic risk profile among Yup’ik Alaska Native people. Ethn Health. 2017;1–15. 10.1080/13557858.2017.1280136 PMC5796859 28116909

[53] Fischer R, Clair C, Studer J, et al Prevalence and factors associated with use of smokeless tobacco in young Swiss men. Eur J Public Health. 2014;24:459–464. 23813708 10.1093/eurpub/ckt086

[54] Omole OB, Ogunbanjo GA Smokeless tobacco: is it really safe? South Afr Fam Pract. 2014;51:292–295.

[55] Grandner MA, Schopfer EA, Sands-Lincoln M, et al The relationship between sleep duration and body mass index depends on age. Obesity. 2015;23:2491–2498. 26727118 10.1002/oby.21247 PMC4700549

[56] Kaufman A, Augustson EM, Patrick H Unraveling the relationship between smoking and weight: the role of sedentary behavior. J Obes. 2012;2012:11. 10.1155/2012/735465 PMC3180774 21961058

[57] Skrypnik D, Bogdański P, Mądry E, et al Effects of endurance and endurance strength training on body composition and physical capacity in women with abdominal obesity. Obesity Facts. 2015;8:175–187. 25968470 10.1159/000431002 PMC5652894

[58] Skrypnik D, Ratajczak M, Karolkiewicz J, et al Effects of endurance and endurance-strength exercise on biochemical parameters of liver function in women with abdominal obesity. Biomed Pharmacother. 2016;80:1–7. 27133033 10.1016/j.biopha.2016.02.017

[59] Szulińska M, Skrypnik D, Ratajczak M, et al Effects of endurance and endurance-strength exercise on renal function in abdominally obese women with renal hyperfiltration: a prospective randomized trial. Biomed Environ Sci. 2016;29:706–712. 27927270 10.3967/bes2016.095

[60] Dumesnil C, Dauchet L, Ruidavets JB, et al Alcohol consumption patterns and body weight. Ann Nutr Metabolism. 2013;62:91–97. 10.1159/000342839 23327878

[61] Liangpunsakul S, Crabb DW, Qi R Relationship between alcohol intake, body fat, and physical activity – a population-based study. Ann Epidemiol. 2010;20:670–675. 20696406 10.1016/j.annepidem.2010.05.014 PMC2921229

[62] Addolorato G, Capristo E, Marini M, et al Body composition changes induced by chronic ethanol abuse: evaluation by dual energy X-ray absorptiometry. Am J Gastroenterol. 2000;95:2323–2327. 11007236 10.1111/j.1572-0241.2000.02320.x

[63] Decherf S, Demeneix BA The obesogen hypothesis: a shift of focus from the periphery to the hypothalamus. J Toxicol Environ Health B Crit Rev. 2011;14:423–448. 21790320 10.1080/10937404.2011.578561

