# Supplementary material for: Socio-demographic and behavioural determinants of body mass index among an adult population in rural Northern Ghana: the AWI-Gen study
Source: Glob Health Action. 2018 Jul 11;11(Suppl 2):1467588. doi: 10.1080/16549716.2018.1467588 (PMC6041816; doi:10.1080/16549716.2018.1467588)
Supplement: Supplemental Material [file ZGHA_A_1467588_SM3241.docx]

**Appendix: Supplementary material**

**Table S1a:** Bivariable analysis of factors (socio-demographic) associated with BMI among adult women and men in Navrongo

|  | **Women** | | **Men** | | **Total** | |
| --- | --- | --- | --- | --- | --- | --- |
|  | **β-Coefficient (95% CI)** | ***p* - value** | **β-Coefficient (95% CI)** | ***p* - value** | **β-Coefficient (95% CI)** | ***p* - value** |
| **Socio-demographic factors** | | | | | |  |
| **Age (in years)** | -0.005 (-0.006, -0.003) | < 0.0001 | -0.004 (-0.006, -0.003) | < 0.0001 | -0.004 (-0.005, -0.003) | < 0.0001 |
| **Ethnicity**  Kasenna  Nankana  Others | ref  -0.080 (-0.099, -0.060)  0.023 (-0.014, 0.059) | ref  < 0.0001 | Ref  -0.062 (-0.079, -0.044)  0.103 (0.040, 0.166) | ref  < 0.0001 | ref  -0.072 (-0.086, 0.059)  0.054 (0.024, 0.085) | ref  < 0.0001 |
| **Number of siblings** | 0.003 (0.001, 0.005) | < 0.0001 | 0.002 (0.001, 0.037) | 0.008 | 0.002 (0.001, 0.003) | < 0.0001 |
| **Parity** | -0.007 (-0.013, -0.002) | 0.008 | - | - | - | - |
| **Partner status** |  |  |  |  |  |  |
| Never married  Currently married  Divorced/Separated | ref  -0.106 (-0.249, 0.036)  -0.133 (-0.276, 0.010) | ref  0.045 | ref  0.068 (-0.003, 0.141)  0.023 (-0.053, 0.098) | ref  0.061 | ref  0.037 (-0.031, 0.106)  0.023 (-0.047, 0.092) | ref  0.186 |
| **Highest education completed**  No formal education  Primary  Secondary  Tertiary | ref  0.045 (0.019, 0.070)  0.113 (0.070, 0.155)  0.277 (0.173, 0.381) | ref  < 0.0001 | ref  0.020 (-0.002, 0.042)  0.032 (0.004, 0.059)  0.115 (0.062, 0.168) | ref  < 0.0001 | ref  0.024 (0.007, 0.042)  0.044 (0.020, 0.068)  0.138 (0.087, 0.188) | ref  < 0.0001 |
| **Employment status**  Employed  Unemployed | ref  -0.596 (-1.063, -0.129) | ref  0.012 | ref  -0.271 -0.701, 0.159) | ref  0.162 | ref  -0.389 (-0.715, -0.064) | ref  0.019 |
| **Household SES**  Poorest  Very poor  Poor  Less poor  Least poor | ref  -0.014 (-0.043, 0.015)  0.015 (-0.014, 0.044)  0.030 (0.003, 0.059)  0.132 (0.102, 0.163) | ref  0.030 | ref  -0.021 (-0.052, 0.010)  0.022 (-0.008, 0.053)  0.011 (-0.017, 0.039)  0.089 (0.060, 0.117) | ref  0.005 | ref  -0.018 (-0.040, 0.003)  0.016 (-0.005, 0.037)  0.017 (-0.003, 0.038)  0.099 (0.079, 0.120) | ref  < 0.0001 |
| **People-to-bedroom density** | 0.007 (-0.002, 0.016) | 0.109 | 0.150 (0.007, 0.023) | < 0.0001 | 0.012 (0.006, 0.018) | < 0.0001 |

Table S1b: Bivariable analysis of behavioural factors associated with BMI among adult women and men in Navrongo

|  | **Women** |  | **Men** |  | **Total** |  |
| --- | --- | --- | --- | --- | --- | --- |
|  | **β-Coefficient (95% CI)** | ***p* - value** | **β-Coefficient (95% CI)** | ***p* - value** | **β-Coefficient (95% CI)** | ***p* - value** |
| **Behavioural factors** |  |  |  |  |  |  |
| **Smoking status**  Never smoked  Former smoking  Current smoking | ref  -0.090 (-0.160, -0.020)  -0.059 (-0.139, 0.020) | ref  0.012 | ref  -0.069 (-0.089, -0.049)  -0.023 (-0.047, 0.001) | ref  < 0.0001 | ref  -0.087 (-0.103, -0.069)  -0.041 (-0.063, -0.019) | ref  < 0.0001 |
| **Smokeless tobacco**  **No**  **Yes** | ref  -0.076 (-0.107, -0.045) | ref  < 0.0001 | ref  -0.006 (-0.036, 0.024) | ref  0.697 | ref  -0.044 (-0.066, -0.022) | ref  < 0.0001 |
| **Alcohol consumption**  Never consumed  Previous consumption  Current non-problematic^[[1]](#footnote-1)^  Current problematic | 0.049 (0.021, 0.077)  ref  0.022 (-0.005, 0.050)  -0.010 (-0.034, -0.013) | 0.001  ref | 0.073 (0.039, 0.108)  ref  -0.027 (-0.047, -0.006)  -0.003 (-0.031, -0.026) | < 0.0001  ref | 0.059 (0.037, 0.082)  ref  -0.026 (-0.042, -0.010)  -0.007 (-0.025, -0.012) | < 0.0001  ref |
| **Exposure to pesticide**  No  Yes | ref  0.044 (0.024, 0.063) | ref  < 0.0001 | ref  0.054 (0.035, 0.072) | ref  < 0.0001 | ref  0.042 (0.028, 0.055) | ref  < 0.0001 |
| **Dietary history** | | | | | |  |
| Fruit servings per day | 0.002 (-0.004, 0.008) | 0.495 | 0.006 (0.001, 0.012) | 0.028 | 0.004 (0.001, 0.008) | 0.042 |
| Vegetable servings per day | -0.004 (-0.011, 0.022) | 0.198 | -0.004 (-0.009, 0.002) | 0.236 | -0.005 (-0.009, -0.001) | 0.031 |
| Sugar sweetened beverages (SSB) | 0.037 (0.021, 0.053) | < 0.0001 | 0.028 (0.016, 0.041) | < 0.0001 | 0.029 (0.019, 0.039) | < 0.0001 |
| **Physical activity** | | | | | |  |
| MVPA | -0.016 (-0.074, 0.248) | 0.593 | -0.016 (-0.063, 0.429) | 0.695 | -0.042 (-0.082, -0.021) | 0.039 |
| Sedentary time | 0.002 (0.001, 0.035) | < 0.0001 | 0.005 (0.027, 0.007) | 0.009 | 0.003 (0.002, 0.039) | < 0.0001 |
| Average sleep in hours per day | -0.017 (-0.024, -0.009) | < 0.0001 | -0.010 (-0.017, -0.003) | 0.004 | -0.009 (-0.014, -0.004) | < 0.0001 |

Drinking is defined as problematic if there are two or more “yes” answers to the 4 CAGE questions on alcohol consumption [[33](#_ENREF_32)]

Table S2a: Models 1, 2 & 3 of the hierarchical multivariable linear regression analysis for determining factors associated with logBMI among women

| **Variables** | **Adjusted β-coefficients [95%CI]** | ***p*-value** |
| --- | --- | --- |
| **Model 1** | | |
| **Age** | -0.004 [-0.005, -0.002] | **< 0.001** |
| **Ethnicity** |  |  |
| Kassena | ref | ref |
| Nankana | -0.073 [-0.093, -0.054] | **< 0.001** |
| Others | 0.011 [-0.024, 0.047] |  |
| **Partnership Status** |  |  |
| Single | 0.035 [-0.098, 0.167] | 0.607 |
| Currently married | Ref | ref |
| Divorced/Separated | -0.004 [-.019, 0.019] |  |
| **Educational Status** |  |  |
| No formal education | ref | ref |
| Primary education | 0.022 [-0.004, 0.047] | **0.001** |
| Secondary education | 0.067 [0.022, 0.112] |  |
| Tertiary education | 0.174 [0.052, 0.296] |  |
| **Household SES** |  |  |
| Poorest | ref | ref |
| Very poor | -0.008 [-0.037, 0.021] | **< 0.001** |
| Poor | 0.017 [-0.011, 0.047] |  |
| Less poor | 0.037 [0.008, 0.065] |  |
| Least poor | 0.109 [0.077, 0.141] |  |
| **Employment status** |  |  |
| Employed | ref | ref |
| Unemployed | -0.007 [-0.027, 0.012] | 0.439 |
| **People-to-bedroom density** | 0.007 [-0.001, 0.016] | 0.096 |
| **Parity** | 0.002 [-0.019, 0.019] | 0.938 |
| **Number of siblings** | 0.001 [-0.001, 0.003] | 0.079 |
| **Model 2** | | |
| **Smoking History** |  |  |
| Never | ref | ref |
| Previous | -0.193 [-0.399, 0.012] | 0.177 |
| Current | 0.018 [-0.113, 0.149] |  |
| **Smokeless tobacco** |  |  |
| No | ref | ref |
| Yes | -0.039 [-0.069, -0.009] | **0.009** |
| **Alcohol Consumption** |  |  |
| Never | 0.010 [-0.016, 0.036] | 0.306 |
| Previous | ref | ref |
| Current Non-problematic | 0.011 [-0.018, 0.039] |  |
| Current problematic | -0.015 [-0.039, 0.010] |  |
| **Pesticide use** |  |  |
| No | ref | ref |
| Yes | 0.009 [-0.010, 0.029] | 0.350 |
| **MVPA** | 1.370 [-4.510, 7.250] | 0.648 |
| **Sedentary time** | 0.004 [0.001, 0.029] | **0.040** |
| **Average Sleep duration** | -0.008 [0.015, -0.001] | **0.022** |
| **Daily fruit servings** | -0.003 [-0.009, 0.003] | 0.346 |
| **Daily vegetable serving** | -0.002 [-0.009, 0.005] | 0.647 |
| **Sugar sweetened beverages** | 0.012 [-0.003, 0.027] | 0.128 |
| **Model 3** | | |
| **Menopausal Status** |  |  |
| Premenopausal | ref | ref |
| Peri-menopausal | -0.025 [-0.096, 0.045] | 0.646 |
| Post-menopausal | -0.030 [-0.125, 0.065] |  |

Model 1: Adjusted for only socio-demographic factors associated with logBMI; Model 2: Adjusted for behavioural factors associated with logBMI with the inclusion of socio-demographic factors; Model 3: Adjusted for biological factors (menopause) with the inclusion of factors in model 1 and 2.

Table S2b: Models 1 & 2 of the hierarchical multivariable linear regression analysis for determining factors associated with logBMI among men

| **Variables** | **Adjusted β-coefficients [95%CI]** | ***p*-value** |
| --- | --- | --- |
| **Model 1** | | |
| **Age** | -0.003 [-0.004, -0.001] | **< 0.001** |
| **Ethnicity** |  |  |
| Kassena | ref | ref |
| Nankana | -0.050 [-0.068, -0.033] | **< 0.001** |
| Others | 0.075 [0.016, 0.134] |  |
| **Partnership Status** |  |  |
| Single | -0.063 [-0.129, 0.003] | **0.005** |
| Currently married | ref | ref |
| Divorced/Separated | -0.037 [-0.063, -0.011] |  |
| **Educational Status** |  |  |
| No formal education | ref | ref |
| Primary education | 0.005 [-0.016, 0.026] | **0.028** |
| Secondary education | 0.008 [0.005, 0.039] |  |
| Tertiary education | 0.074 [0.022, 0.126] |  |
| **Household SES** |  |  |
| Poorest | ref | ref |
| Very poor | -0.011 [-0.042, 0.017] | **< 0.001** |
| Poor | 0.025 [-0.004, 0.054] |  |
| Less poor | 0.008 [-0.019, 0.036] |  |
| Least poor | 0.072 [0.043, 0.101] |  |
| **Employment Status** |  |  |
| Employed | ref | ref |
| Unemployed | 0.002 [-0.016, 0.021] | 0.793 |
| **People-to-bedroom density** | 0.007 [-0.005, 0.015] | 0.068 |
| **Number of siblings** | 0.001 [-0.004, 0.002] | 0.223 |
| **Model 2** | | |
| **Smoking History** |  |  |
| Never | ref | ref |
| Previous | -0.092 [-0.158, -0.026] | **< 0.001** |
| Current | -0.216 [-0.097, 0.054] |  |
| **Smokeless tobacco use** |  |  |
| No | ref | ref |
| Yes | 0.007 [-0.020, 0.036] | 0.611 |
| **Alcohol Consumption** |  |  |
| Never | 0.008 [-0.031, 0.048] | **0.001** |
| Previous | ref | ref |
| Current Non-problematic | -0.005 [-0.035, -0.001] |  |
| Current problematic | -0.021 [-0.042, -0.003] |  |
| **Pesticide use** |  |  |
| No | ref | ref |
| Yes | 0.022 [0.003, 0.042] | **0.022** |
| **MVPA** | 0.221 [-0.343, 0.785] | 0.443 |
| **Sedentary time** | 0.045 [0.019, 0.074] | **0.026** |
| **Average Sleep duration** | -0.002 [-0.004, -0.001] | **0.018** |
| **Daily fruit servings** | -0.003 [-0.008, 0.003] | 0.349 |
| **Daily vegetable serving** | 0.002 [-0.006, 0.007] | 0.948 |
| **Sugar sweetened beverages** | 0.014 [-0.009, 0.029] | 0.066 |

Model 1: Adjusted for only socio-demographic factors associated with logBMI; Model 2: Adjusted for behavioural factors associated with logBMI with the inclusion of socio-demographic factors

Table S3: Operational definition of some of the variables used in this paper

| **Variables** | **Operational definition** |
| --- | --- |
| Smoking history | Defined as never smoked, previously smoked and currently smoking at least one cigarette per day |
| Smokeless tobacco | Smokeless tobacco use referred to snuff users and those who chew tobacco. |
| Alcohol consumption | Alcohol consumption was categorized into never, previous intake, and current non-problematic and problematic intake. A participant is said to have a current problematic drinking pattern if they answered yes to more than two of the following questions on the CAGE questionnaire on alcoholism [[34](#_ENREF_32)] which was also a component of the main AWI-Gen questionnaire:   1. Have you ever felt that you should cut down on your drinking? 2. Have people annoyed you by criticizing your drinking? 3. Have you ever felt bad or guilty about your drinking? 4. Have you ever had an alcoholic drink first thing in the morning to steady your nerves or get rid of a hangover? |
| Physical activity | The global physical activity questionnaire (GPAQ) used to assess self-reported physical activity [[33](#_ENREF_31)] was a component of the main AWI-Gen questionnaire. The total moderate-vigorous physical activity (MVPA) in minutes per week was calculated from the accumulated occupation, travel-related and leisure time physical activity |
| Crowding (Person-to-bedroom density) | Crowding in a home was measured using person-to-bedroom density which was calculated by dividing the number of persons in a household by the number of bedrooms. |
| Sugar Sweetened Beverages (SSB) | Defined as the number of cans, bottles, or cups of sugary drinks such as Coke, Pepsi, Fanta, Sprite, etc consumed per day. |
| Average sleep duration in hours per day | Sleep duration was self reported and assessed through the question "What time do you go to sleep and what time do you wake up during the week and on weekends". Average sleep duration was then defined as an average of the number of hours spent sleeping during the week and during the weekends. |
| Menopausal status | Menopausal status was categorised as   - Pre-menopausal where participants reported normal periods over the past year; - Peri-menopausal where participants have irregular periods over the past year and - Post-menopausal where participants reported no periods within the past year. |

1. [↑](#footnote-ref-1)
